# Comparative Analysis of Meat Quality in Hu Sheep and Their Crossbred Lambs

**DOI:** 10.3390/foods15081444

**Published:** 2026-04-21

**Authors:** Lei Zhang, Shuwei Dong, Yujia Xing, Siqi Li, Shutao Shang, Zhihao Wang, Shijie Bi, Fenghong Wang, Gao Gong, Lei Qu

**Affiliations:** 1 College of Advanced Agricultural, Yulin University, Yulin 719000, China; nmgzhangl@126.com (L.Z.);; 2 Shaanxi Provincial Engineering and Technology Research Center of Cashmere Goats, Yulin University, Yulin 719000, China; nmgcfwfh@163.com; 3 College of Food Science and Pharmacy, Xinjiang Agricultural University, Urumqi 830052, China; 4 College of Animal Sciences, Xinjiang Agricultural University, Urumqi 830052, China

**Keywords:** Hu sheep, lamb, meat quality, flavor

## Abstract

This study focuses on the selection of hybrid combinations of Hu sheep and meat quality analysis. A comparative analysis of meat quality and volatile flavor compounds was conducted using three hybrid groups—Australian White–Hu (AH), White Suffolk–Hu (SH), and Southdown–Hu (NH)—and a pure Hu sheep group (HH) as research subjects. The results show that in terms of basic nutritional quality, the moisture content in the NH group was significantly higher than that in the HH group (*p* < 0.05), and the crude protein content in the NH group was significantly higher than that in the HH group (*p* < 0.05). Regarding physicochemical properties, the NH group had significantly higher meat color scores, L*, a*, and b* values, than the other groups (*p* < 0.05), along with the best tenderness and cooking yield. An analysis of amino acids, fatty acids, and volatile flavor compounds in lambs from different hybrid combinations revealed significant differences in the contents of lys, thr, asp, and his (*p* < 0.01). Although no significant differences were found in the fatty acid composition scores among the AH, SH, NH, and HH groups, all groups met the FAO/WHO recommended values. The NH group not only had the highest MUFA and total fatty acid content but also the highest levels of trans-petroselinic acid and trans-vaccenic acid, the two most abundant trans fatty acids. A total of 43 volatile organic compounds were detected in the four groups, among which 10 were identified as differential compounds. This study provides a scientific basis for the hybrid utilization of Hu sheep and offers technical support for the transformation and upgrading of the regional meat sheep industry.

## 1. Introduction

Lamb meat, valued globally as a significant source of high-quality animal protein due to its high protein content, low fat profile, and distinctive flavor, consistently enjoys widespread consumer preference [1]. In recent years, sustained economic development and a marked improvement in residents’ living standards have driven a steady increase in total lamb consumption [2]. Consequently, the lamb industry plays an increasingly crucial role in safeguarding national nutrition and health, boosting income for farmers and herders, and fostering regional economic prosperity [3].

The Hu sheep, a unique and excellent indigenous sheep breed resource in China, is renowned for its exceptional reproductive performance, strong maternal instincts, excellent lactation capacity, and high adaptability to confined housing environments [4]. It constitutes a valuable genetic resource pool. Its potential as a dam is particularly outstanding, providing a solid foundation for establishing efficient hybrid production systems [5]. Practical evidence demonstrates that the scientific introduction of foreign meat-type rams, characterized by fast growth rates, superior meat production performance, and desirable meat quality traits, can effectively utilize heterosis [6]. This approach significantly enhances the growth efficiency of hybrid offspring, evidenced by improved daily weight gain and feed conversion ratio, alongside enhanced overall economic performance, such as shortened finishing cycles and improved carcass quality [7]. This strategy represents one of the core technological pathways for rapidly improving China’s mutton sheep production.

Simultaneously, structural upgrades in the consumer market impose higher demands on the lamb industry. Modern consumers have moved beyond basic protein supplementation. There is a growing preference for lamb products that provide enhanced nutritional profiles, including abundant essential amino acids and unsaturated fatty acids. Concurrently, sensory qualities such as milder odor, uniform intramuscular fat distribution, and improved tenderness are also increasingly valued [8]. This shift in consumer preferences renders traditional farming models focused solely on yield increasingly unsustainable. A quality-oriented approach has thus become paramount for the industry’s sustainable development. However, current research predominantly concentrates on growth and slaughter performance, with studies on eating quality remaining relatively scarce.

Against this backdrop, this study focuses on the meat quality evaluation of Hu sheep and their hybrid offspring. The core research involves systematically assessing key meat quality indicators of lambs produced from various crossbreeding combinations between introduced elite foreign meat-type rams (sires) and Hu sheep (dams) (Southdown × Hu, Suffolk × Hu, and Australian White × Hu). These indicators include meat color, pH values, shear force, drip loss, cooking loss, intramuscular fatty acid content, and the composition of key flavor compounds. The primary objective is to identify the optimal crossbreeding combination for lamb meat eating quality under specific feeding and management conditions through scientifically rigorous comparative experiments and analysis.

## 2. Materials and Methods

### 2.1. Materials

This experiment was conducted at the sheep farm of Bafu Eco-Agriculture Co., Ltd., in Dingbian County, Yulin City. Healthy multiparous Hu ewes were selected and subjected to estrus synchronization using progesterone intravaginal sponges. The composition of the basal concentrate diet was as follows: corn, 56.4%; soybean meal, 30.0%; wheat bran, 6.0%; salt, 1.0%; sodium bicarbonate, 1.0%; limestone powder, 1.6%; and premix, 4.0%. The nutrient levels of the diet (on a dry matter basis) were: crude protein, 15.32%; crude ash, 8.66%; ether extract, 1.69%; neutral detergent fiber, 17.33%; acid detergent fiber, 12.47%; calcium, 1.20%; and phosphorus, 0.48%. Artificial insemination was performed using semen from Australian White, White Suffolk, Southdown, and Hu rams. Lambs from each group were weighed at birth, and at weaning (2 months of age), 30 lambs per group (15 males and 15 females, totaling 120 lambs) were randomly selected based on similar body weight and age. These were designated as: the Australian White × Hu crossbred group (AH group), the White Suffolk × Hu crossbred group (SH group), the Southdown × Hu crossbred group (NH group), and the Hu × Hu crossbred group (HH group). Following weaning, all lambs were fed for 4 months and slaughtered at 6 months of age, with a body weight ranging from 43 to 45 kg.

### 2.2. Sample Preparation

Samples of the *longissimus dorsi* muscle (approximately 500 ± 20 g) between the 12th and 13th ribs of the left carcass and bilateral psoas major muscles (approximately 100 ± 5 g) were collected from each experimental sheep. The samples were aseptically placed into food-grade vacuum-sealed bags, transported under refrigerated conditions to the Laboratory of the Modern College of Agriculture at Yulin University, and stored at −80 °C, pending analysis.

### 2.3. Analysis of Basic Nutritional Components

A total of 100 muscle samples were collected from the *longissimus dorsi* muscle, specifically the portion 20–30 cm posterior to the 3rd–4th thoracic vertebrae, at 45 min postmortem. The pH value of each muscle sample was determined using a precision pH meter. Moisture, crude fat, and crude protein contents were measured respectively according to previous research conducted by Wang et al. [9].

### 2.4. Analysis of Color

Color parameters (L*, a*, and b*) were measured in triplicate using a CR-400 colorimeter (Zhuoxie Instrument Equipment Co., Ltd., Guangzhou, China) under the following conditions: blooming time, 30 min; sample temperature, 4 °C; measurement position; and shear force core size, 1 cm × 1 cm × 5 cm. L signifies lightness (0 = black, 100 = white), a represents the red–green axis (−60 = green, +60 = red), and b corresponds to the yellow–blue axis (−60 = blue, +60 = yellow).

### 2.5. Analysis of Cooking Loss Rate

The cooking loss was determined according to the method described by Leal et al. with slight modifications [10]. Samples were weighed (M_1_, g) and placed in cooking bags, then immersed in a 100 °C water bath for 20 min until the core temperature reached 70 °C. The meat samples were removed and cooled at room temperature (20–22 °C) for 30 min. The surface moisture was blotted, and the samples were reweighed (M_2_, g). The cooking loss (R) was calculated using the following formula:

$$R(\%)=(M_{1}-M_{2})/M_{1}\times 100\%$$

### 2.6. Analysis of Water Loss Rate

The water loss rate was also determined according to the method described by Leal et al. with slight modifications [10]. Fresh lamb samples were weighed (W_1_, g) and placed in centrifuge tubes. Samples were centrifuged at 1000× *g* for 10 min at 4 °C. After centrifugation, samples were removed, the surface moisture was gently blotted with filter paper, and the samples were reweighed (W_2_, g). The water loss rate (Y) was calculated using the following formula:

$$Y(\%)=(W_{1}-W_{2})/W_{1}\times 100\%$$

### 2.7. Analysis of Tenderness

The shear force of *longissimus dorsi* muscle samples was measured using a TMS-RO texture analyzer (Beijing Yingsheng Hengtai Technology Co., Ltd., Beijing, China). For each of 3 to 5 meat pieces, the shear force value was recorded individually, and the arithmetic mean was then calculated.

### 2.8. Analysis of Amino Acids

The determination of amino acids was conducted according to the method described by Ji et al. [11]. Quantitative analysis of amino acids was conducted using an ultra-high performance liquid chromatography–tandem mass spectrometry (UHPLC-MS/MS) system comprising an ExionLC™ AD UHPLC (Applied Biosystems, Boston, MA, USA) coupled to a Triple Quad™ 6500+ mass spectrometer (SCIEX, Framingham, MA, USA). Chromatographic separation was achieved on a Waters ACQUITY UHPLC BEH Amide column (2.1 mm × 100 mm, 1.7 μm) maintained at 50 °C, employing a mobile phase of solvent A (5 mmol/L of ammonium acetate with 0.1% formic acid) and solvent B (acetonitrile with 0.1% formic acid) at 0.30 mL/min flow rate. The gradient program was set as follows: 80% B (0–0.5 min), linear decrease to 70% B (0.5–2.5 min), further decrease to 45% B (2.5–6.5 min), rapid increase to 80% B (6.5–6.6 min), and hold until 9.0 min. Mass spectrometric detection operated in positive electrospray ionization (ESI) mode with multiple reaction monitoring (MRM), using optimized parameters: ion spray voltage, 5500 V; curtain gas, 35 psi; temperature, 550 °C; nebulizer gas, 50 psi; and heater gas, 60 psi.

### 2.9. Analysis of Fatty Acids

The determination of fatty acids was performed with reference to Yi et al.’s method with minor modifications [12]. Quantitative analysis was performed using an ultra-high performance liquid chromatography–tandem mass spectrometry (UHPLC-MS/MS) system comprising an ExionLC™ AD UHPLC coupled to a Triple Quad™ 6500+ mass spectrometer. Chromatographic separation was conducted on a Waters ACQUITY UPLC BEH C18 column (2.1 mm× 100 mm, 1.7 μm) maintained at 40 °C, with a mobile phase consisting of solvent A (acetonitrile/water, 1:1, *v*/*v*, containing 0.1% formic acid) and solvent B (isopropanol/acetonitrile, 1:1, *v*/*v*) at a flow rate of 0.30 mL/min. The gradient program was set as follows: 45% B (0–1.0 min), linear increase to 70% B (1.0–5.5 min), further increase to 75% B (5.5–14.5 min), progression to 80% B (14.5–27.0 min), rise to 100% B (27.0–41.0 min), rapid decrease to 45% B (41.0–42.1 min), and hold until 44.0 min. Mass spectrometric detection was operated in negative electrospray ionization (ESI) mode with multiple reaction monitoring (MRM) using optimized parameters: ion spray voltage, −4500 V; curtain gas, 35 psi; source temperature, 550 °C; and both nebulizer gas and heater gas, 60 psi.

### 2.10. Analysis of VOCs by GC-IMS

The determination of VOCs was performed with reference to Wang et al.’s method with minor modifications [13]. Sample preparation involved homogenizing meat samples to a uniform mince using a grinder, followed by precise weighing of 2.0 g aliquots into 20 mL headspace vials, ensuring even distribution. Analysis was conducted via an automated headspace sampler with these parameters: a 60 °C equilibration temperature for 15 min under 50 rpm agitation, an 85 °C injection needle temperature, a 500 μL injection volume, and splitless injection mode. Ion Mobility Spectrometry (IMS) conditions featured a constant 45 °C temperature with high-purity nitrogen (>99.99%) as drift gas at 150 mL/min. For GC analysis, separation employed an MXT-5 column (15 m × 0.53 mm, 1.0 μm; RESTEK, Bellefonte, PA, USA) maintained isothermally at 60 °C for 20 min, using high-purity nitrogen (>99.99%) carrier gas with constant 150 mL/min drift gas flow. The GC column flow program was: 2 mL/min (0–2 min), linear ramp to 10 mL/min (2–10 min), then linear increase to 100 mL/min (10–20 min) with a 10 min hold. Following 15 min of headspace incubation at 60 °C, vapor components were aspirated by the 85 heated syringe and injected into the FlavourSpec^®^ flavor analyzer (G.A.S., Dortmund, Germany) for volatile compound detection.

### 2.11. Statistical Analysis

Statistical analysis was performed using one-way ANOVA, with post hoc LSD tests identifying intergroup differences (significance threshold: *p* < 0.05). Volatile organic compounds (VOCs) were characterized using the GC × IMS Library Search software (VOCal 0.4.03 rev300) (G.A.S., Dortmund, Germany).

## 3. Results

### 3.1. Changes in Basic Nutritional Components

**Figure 1:**
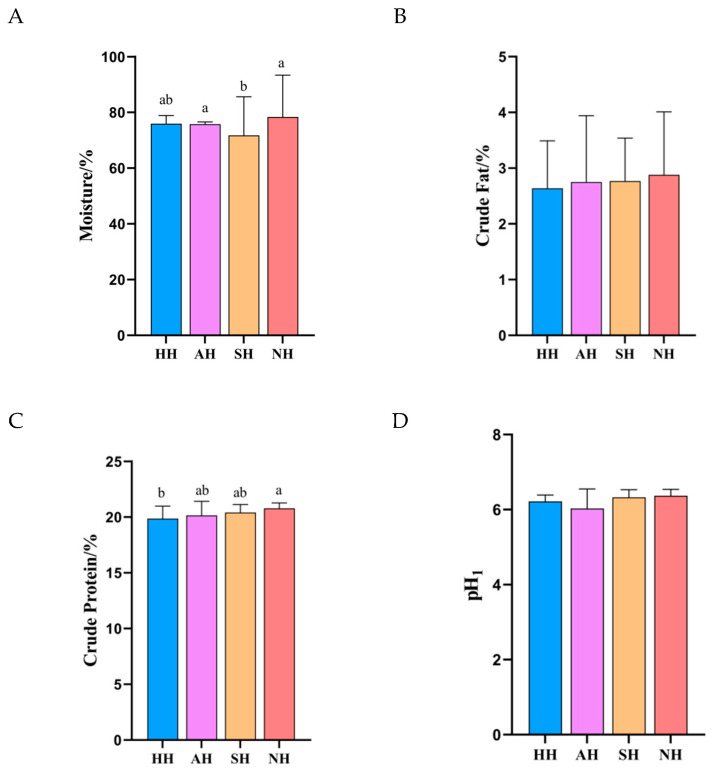
Basic nutritional components of crossbred lambs of different meat sheep breeds and Hu sheep. (**A**) Moisture, (**B**) crude fat, (**C**) crude protein, and (**D**) pH. Different small letters within the same row denote significant differences between means at *p* < 0.05.

Muscle nutritional composition constitutes a critical determinant of eating quality. Its water dynamics, governed by water holding capacity, directly influence meat color stability, tenderness, and flavor release efficiency [14]. This study demonstrated that moisture content across four lamb groups ranged from 71.74% to 78.39% (Figure 1A). The NH group exhibited significantly higher moisture content (76.82 ± 0.31%) than the HH group (73.15 ± 0.42%) (*p* < 0.05). This divergence likely originates from distinct myofibrillar protein conformations.

Moderate intramuscular fat content significantly enhanced meat juiciness, improved textural properties, and increased tenderness, thereby elevating overall eating quality [15]. Conversely, excessive fat induced textural deterioration and produced a greasy mouthfeel. In the present study, all four lamb groups exhibited crude fat concentrations within this optimal range (2–3%, Figure 1B), indicating superior eating quality characterized by desirable tenderness, juiciness, and palatability. These attributes suggest potential for delivering a premium culinary experience to consumers.

The nutritional value of meat is largely determined by its protein content. As an essential core nutrient in meat, protein level serves as a key indicator for evaluating nutritional quality [16]. Being a fundamental component of human tissues, protein’s nutritional value is intrinsically linked to muscle tissue characteristics. As shown in Figure 1C, significant differences (*p* < 0.05) were observed in crude protein content among the four experimental groups (HH, AH, SH, and NH). Compared with the HH group, protein content increased by 1.46%, 2.77%, and 4.68% in the AH, SH, and NH groups, respectively. Notably, the NH group exhibited significantly higher protein content than the HH group (*p* < 0.05). These results indicate that NH sheep contain elevated protein levels, suggesting superior nutritional value.

pH value serves as a critical indicator of lamb meat quality, directly influencing color, water-holding capacity, tenderness, and shelf life [17]. The optimal pH range at 45 min post-slaughter should fall between 6.0 and 6.7. Severe pre-slaughter stress may result in elevated glycogen reserves, potentially causing a rapid pH decline during the early post-slaughter phase within 1–3 h. Under such conditions, the pH can drop to suboptimal levels below 6.0 at 45 min post-slaughter. When muscle temperature remains elevated, a swift pH reduction to the isoelectric point of proteins near 5.2–5.5 triggers irreversible denaturation and contraction of myofibrillar proteins, accompanied by a sharp deterioration in water-holding capacity. These biochemical changes manifest as pale, soft, exudative meat characterized by pale coloration, soft texture, excessive exudate, substantial drip loss, reduced tenderness, and coarse texture. In the present study, all four experimental groups exhibited pH values of approximately 6.2, indicating minimized storage losses and superior lamb meat quality (Figure 1D).

### 3.2. Changes in Color

**Figure 2:**
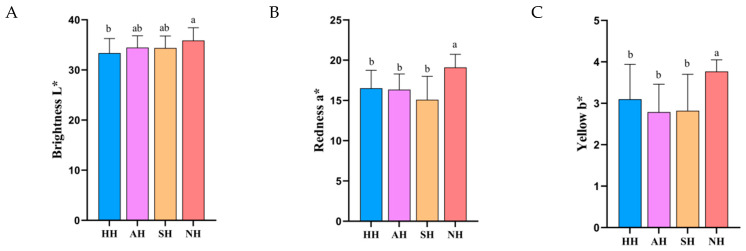
Color of crossbred lambs of different meat sheep breeds and Hu sheep. (**A**) L* value, (**B**) a* value, and (**C**) b* value. Different small letters within the same row denote significant differences between means at *p* < 0.05.

As shown in Figure 2A–C, meat color parameters measured by a chroma meter revealed that the NH group exhibited significantly higher L* values than the HH group (*p* < 0.05), while its a* and b* values were significantly elevated compared to all other three groups (*p* < 0.05). The redness (a* value) demonstrated a positive correlation with the oxymyoglobin state, where higher values indicated more vivid red coloration. Data from this study showed that the NH lamb group achieved significantly greater a* values than the remaining groups (*p* < 0.05), indicating brighter redness. This molecular characteristic enhanced oxygen-binding capacity, thereby improving oxygen utilization efficiency during metabolic processes [18]. Concurrently, the South Hu lamb group displayed a significantly increased L* value compared to the Hu lamb group (*p* < 0.01), with its b* value also being significantly higher than all control groups (*p* < 0.05). According to meat color science principles, the elevated L* and b* values collectively indicated inhibited myoglobin oxidation, demonstrating stronger oxidative stability in NH lamb muscle tissue [19,20]. This biochemical property not only contributed to superior surface gloss but also implied lower lipid oxidation levels, which may enhance color stability during storage.

### 3.3. Changes in Cooking Loss Rate, Water Loss Rate and Tenderness

**Figure 3:**
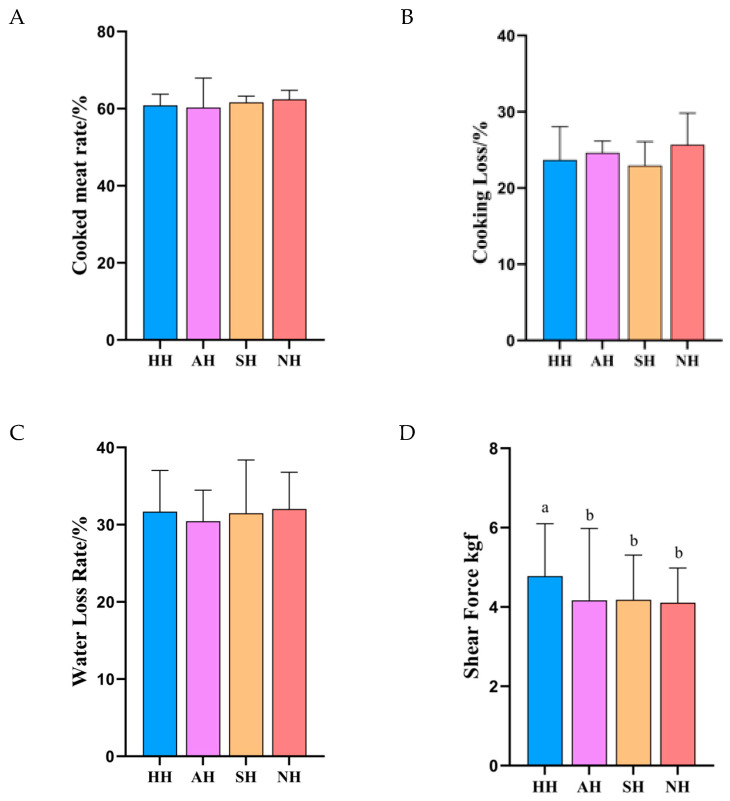
Edible quality of crossbred lambs of different meat sheep breeds and Hu sheep. (**A**) Cooked meat rate, (**B**) cooking loss, (**C**) water loss rate, and (**D**) shear force. Different small letters within the same row denote significant differences between means at *p* < 0.05.

Cooking yield served as a core indicator of cooking loss in muscle tissue. A higher cooking yield denoted superior water-holding capacity during heating [21]. As shown in Figure 3A, all four lamb groups exhibited cooking yields approximating 60%, indicating comparable cooking losses across samples. Concurrently, elevated thermal denaturation temperatures diminish cooking loss (Figure 3B). These findings collectively indicate superior tenderness and juiciness in NH lamb meat. Cooking loss (Figure 3B) demonstrated a positive correlation with water loss rate, both reflecting moisture retention capabilities. Water loss rate constituted a critical quality parameter for evaluating water-holding properties, directly mirroring muscle cells’ capacity to maintain moisture equilibrium [22]. Figure 3C reveals non-significant differences (*p* > 0.05) in water loss rates among the four lamb groups. Shear force functioned as a key metric for tenderness evaluation [23]. Tenderness and juiciness, as primary sensory attributes, collectively determined meat eating quality [24]. Previous studies confirmed that breed is the predominant factor influencing tenderness, with significant variation existing among breeds within the same species [25]. Enhanced hydrophilicity in the myosin heads of NH lambs contributes to expanded intermyofibrillar spacing, which reduces shear force values, as validated in Figure 3D. As presented in Figure 3D, AH, SH, and NH lambs displayed significantly lower shear force values (*p* < 0.05) compared to HH, demonstrating superior tenderness. These findings indicate that crossbreeding enhances meat quality by improving palatability and facilitating mastication.

### 3.4. Changes in Amino Acids

Meat and meat products serve as crucial food sources, rich in essential nutrients indispensable to the human body, including amino acids, essential fatty acids, various vitamins, and minerals [26]. These nutrients play a vital role in maintaining normal physiological functions and biochemical metabolic processes [11]. The amino acid composition and content in the *longissimus dorsi* muscle of different breeds of meat sheep and Hu sheep-crossbred lambs analyzed in this study are presented in Table 1. Twenty-three amino acids were detected in the four groups: AH, SH, NH, and HH. Among these, seven were essential amino acids (EAAs) for humans: Lysine, Methionine, Threonine, Valine, Leucine, Isoleucine, and Phenylalanine. Sixteen were non-essential amino acids (NEAAs): Tryptophan, Tyrosine, Aspartic acid, Histidine, Glutamic acid, Glycine, Alanine, Arginine, Serine, Creatine, Proline, Asparagine, Glutamine, γ-Aminobutyric acid, Ornithine, and Taurine. Regarding EAAs, the Lys content in the NH group was significantly higher than that in the HH group (*p* < 0.01). The Threonine content in the HH, SH, and NH groups was significantly higher than that in the AH group (*p* < 0.01).

Methionine and lysine are regarded as the two most critical limiting amino acids for ruminants [27]. Among the four groups, methionine exhibited the highest content. Its most significant role is participation in protein synthesis. Lysine, functioning as both a ketogenic and glucogenic amino acid and serving as the reference amino acid in the ideal amino acid model, participates in physiological processes such as energy metabolism and protein synthesis within the animal body [28]. This involvement is closely associated with animal growth and development. Genetic background differences among different sheep breeds lead to variations in the nutritional composition of their muscles. This suggests that due to the genetic characteristics of their paternal lineage, NH sheep possess more active enzymes involved in lysine anabolism and more efficient mechanisms for lysine absorption and transport.

Threonine is an essential amino acid required for the growth and development of mammals [29]. As an important nutritional fortifier, threonine not only promotes protein synthesis but also provides energy for the organism, participates in fat metabolism, and contributes to maintaining normal growth, development, and nervous system function [30,31]. HH, SH, and NH sheep exhibited higher threonine content, indicating a more efficient capacity for threonine synthesis, transport, and accumulation during intramuscular growth and protein synthesis processes.

Non-essential amino acids primarily participate in the construction and repair of body tissues, such as the renewal of muscles and skin. They can also be converted into other amino acids within the body; can regulate metabolism; and are used to synthesize enzymes, hormones and so on [32]. Notably, glutamine and GABA are vital nutrients for intestinal mucosal cells. They help maintain intestinal mucosal integrity, ensuring efficient nutrient absorption and providing ample nutritional support for bodily growth and development [33]. Glutamic acid is the key component responsible for the taste of umami, contributing to the delicious flavor of mutton and enhancing its rich, mellow taste after cooking [34]. The AH sheep exhibited the highest levels of glutamine and glutamic acid among the four groups. Concurrently, the higher glutamic acid content enhances meat flavor, making it more acceptable to consumers.

When the amino acid pattern of dietary protein closely matches that of human endogenous proteins, the utilization efficiency of essential amino acids by the human body increases [35]. Consequently, the nutritional value of that protein source is higher. According to the Food and Agriculture Organization (FAO) and World Health Organization (WHO) ratio (FAO/WHO), the proportion of essential amino acids to total amino acids (EAAs/TAAs) in high-quality protein typically falls around 40%, and the ratio of essential amino acids to non-essential amino acids (EAAs/NEAAs) should be above 60% [36]. In the present study, the EAA/TAA ratios for all four groups were approximately 50%, and the EAA/NEAA ratios were around 90%. These results indicate that the essential amino acid composition is well-balanced in the mutton from all four groups, qualifying them all as sources of high-quality protein.

### 3.5. Changes in Fatty Acids

Fatty acid profiles of *longissimus dorsi* muscles from different sheep breeds and Hu hybrid lambs (Table 2) revealed 46 fatty acids across all groups: 14 SFAs and 32 UFAs (18 MUFAs and 14 PUFAs). The NH group exhibited significantly higher total fatty acids (410,493.43 ng/g) than HH (*p* < 0.01), with elevated MUFA levels versus SH/HH (*p* < 0.05). SFA composition was dominated by palmitic/stearic acids (85.93–87.07% of total SFAs), while the NH group showed higher decanoic (vs. HH, *p* < 0.01), lauric/arachidic (vs. SH, *p* < 0.01), tridecanoic/heneicosanoic acids (vs. SH, *p* < 0.05), contrasting with SH’s elevated myristic acid (vs. HH, *p* < 0.01) and AH’s increased lignoceric acid (vs. all, *p* < 0.01). MUFAs primarily comprised trans-petroselinic/trans-vaccenic acids (76.07–78.29%), with the NH group demonstrating higher levels of trans-petroselinic (vs. HH, *p* < 0.05), trans-vaccenic (vs. SH/HH, *p* < 0.05), myristoleic, cis-10-pentadecenoic (vs. HH, *p* < 0.01), trans-10-pentadecenoic (vs. SH/HH, *p* < 0.01), and cis-10-heptadecenoic acids (vs. all, *p* < 0.01). Among PUFAs, linoleic/arachidonic acids predominated (78.08–79.70%), with the NH group displaying elevated cis-11,14-eicosadienoic, cis-13,16-docosadienoic (vs. HH, *p* < 0.01), and EPA levels (vs. HH, *p* < 0.05).

Fatty acid composition and content significantly contribute to meat’s sensory properties, nutritional value, and human health implications [37]. As essential chemical constituents of lipids, fatty acids serve as critical aroma compounds or their precursors [38]. Their composition and concentration in muscle tissue profoundly influence nutritional quality and flavor profiles and exert substantial health impacts [39]. Fatty acid metabolic patterns and profiles directly determine meat quality attributes and nutritional characteristics [40]. The identical spectrum of fatty acids detected across all four lamb *longissimus dorsi* muscle groups suggests that Hu sheep crossbreeding may not alter fundamental fatty acid diversity, though significant quantitative differences were observed. The NH group exhibited the highest total fatty acid content, with elevated levels potentially influencing flavor intensity and juiciness—consistent with findings demonstrating NH’s superior tenderness and higher moisture content. Saturated fatty acids (SFAs) carry notable physiological implications: research indicates they may elevate serum lipoprotein cholesterol levels. Excessive SFA intake increases low-density lipoprotein cholesterol and triglycerides, promoting hyperlipidemia, atherosclerosis, and coronary heart disease risk, primarily attributable to lauric, myristic, and palmitic acids. Notably, lauric acid potentiates fat deposition and enhances myristic/palmitic acid accumulation [41]. In this study, the SH lambs demonstrated significantly lower lauric acid content, while the HH group showed markedly reduced myristic acid levels compared to the NH group, indicating that HH lambs and their SH crosses yield meat that is potentially more beneficial for human health.

Monounsaturated fatty acids (MUFAs) play a critical role in preventing atherosclerosis and reducing coronary heart disease risk [42]. In this study, the NH group exhibited higher MUFA content than the HH and SH groups, indicating that crossbreeding enhances lamb quality. Oleic acid and palmitoleic acid were identified as key functional components: oleic acid—recognized as a “safe fatty acid” in nutritional science—significantly correlates with lamb flavor and reduces cholesterol; palmitoleic acid improves meat quality while lowering blood lipids and atherosclerosis risk [43]. Notably, the AH lambs demonstrated elevated oleic acid content, and the NH hybrids showed the highest palmitoleic acid levels, confirming that crossbreeding improves both meat quality and nutritional value.

Polyunsaturated fatty acids (PUFAs) provide essential fatty acids that humans cannot synthesize. They maintain cell membrane fluidity for physiological functions and significantly impact cardiovascular health, inflammatory responses, and neurological functions through antithrombotic, anti-inflammatory, neuroprotective, and cognitive-enhancing effects [44,45]. Docosahexaenoic acid (DHA) constitutes a vital component of neural and retinal phospholipid membranes [42], while eicosapentaenoic acid (EPA) prevents cardiac fibrosis, with studies suggesting maternal EPA/DHA intake mitigates postpartum depression via anti-inflammatory mechanisms [40]. Although no significant intergroup differences in DHA content were observed, the NH lambs showed higher EPA levels than HH.

Notably, trans-petroselinic and trans-vaccenic acids (predominant PUFAs in this study) are trans fatty acids whose excessive consumption may pose health risks [46]. The elevated levels of these compounds in the NH lambs warrant safety concerns. Conversely, docosadienoic acid exerted anti-inflammatory effects by suppressing pro-inflammatory cytokines (IL-6 and IFN-γ) and modulating TNF expression. Significantly higher cis-13,16-docosadienoic and cis-11,14,17-eicosatrienoic acid levels in the NH versus HH lambs demonstrated that crossbreeding enhances fatty acid profiles and anti-inflammatory capacity.

### 3.6. Identification of VOCs

**Figure 4:**
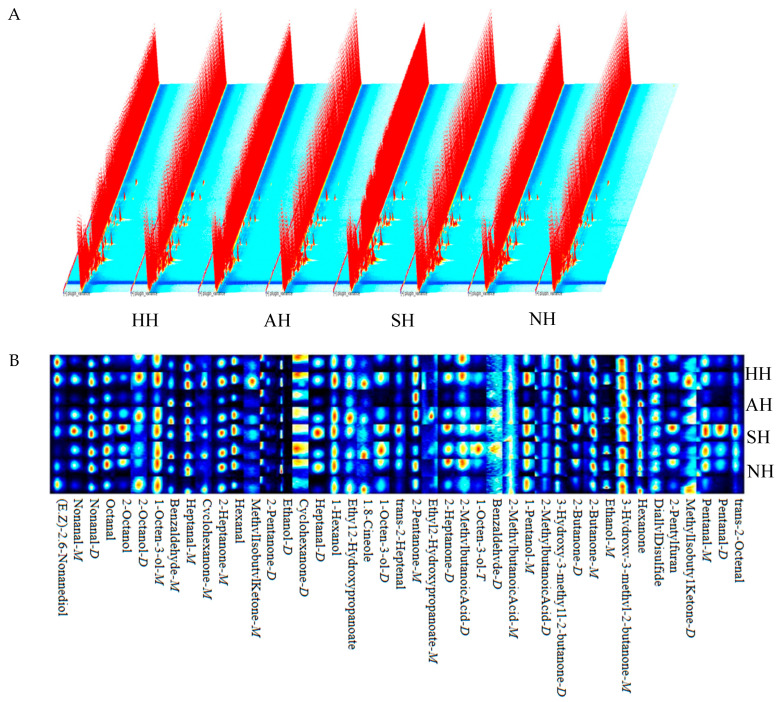
Volatile substances in the mutton of lambs from the crosses between different breeds of mutton sheep and Hu sheep. (**A**) 3D map and (**B**) fingerprint maps.

Volatile organic compounds (VOCs) in the AH lamb, SH lamb, NH lamb, and HH lamb were analyzed using Gas Chromatography–Ion Mobility Spectrometry (GC-IMS). Utilizing the RePorter plugin, the resulting three-dimensional spectral data depicted in Figure 4A were obtained. Within this spectrum, the peak intensities of VOCs varied across the different samples. The distinct red row represents the Reaction Ion Peak (RIP), which is consistently present even in the absence of sample-specific peaks. A visual inspection of Figure 4A readily reveals the emergence of novel signal peaks in the HH and AH samples. However, the SH and NH samples exhibited a greater number of signal peaks with higher intensities. A comparative analysis of the four sample groups indicated discernible differences in peak signal strength, signifying variations in VOC content among the samples. Based on this initial visual assessment, the SH lamb appeared to possess richer diversity and a higher concentration of volatile compounds.

A comparative fingerprint analysis, incorporating all detected peaks, was performed, as shown in the fingerprint plot (Figure 4B). Each row in this plot represents the complete set of selected signal peaks for the VOCs within a specific sample, facilitating a clear visualization of the full VOC profile for each sample type and enabling better discrimination of VOC differences between samples. The fingerprint plot provides a detailed analysis of all signal peaks, where the concentration of VOCs is reflected by the brightness of the color; higher concentrations correspond to increased brightness [47]. The volatile compounds identified across the different lamb breeds encompassed various classes, including alcohols, ketones, aldehydes, esters, acids, ethers, and furans. Figure 4B clearly demonstrates that the SH lamb possessed a significantly greater variety of VOCs compared to the other three samples.

The GC-IMS detection results were compared against a database to conduct a qualitative analysis of the volatile compounds present in the lamb samples. A total of 43 volatile compounds were identified across the four lamb samples, with the specific details presented in Table 3. These compounds comprised 8 aldehydes, 5 alcohols, 8 ketones, 2 esters, 1 acid, 1 furan, and 2 ethers.

The GC-IMS quantitative analysis effectively revealed variations in the concentrations of target compounds among the different samples. As shown in Table 3, the 43 detected VOCs across the four lamb groups exhibited varying abundances. The SH group contained the highest concentrations of flavor-related volatile compounds among the aldehydes, alcohols, ketones, and furans. Notably, the concentration of 2-pentylfuran was significantly higher in the SH group compared to the AH and NH groups (*p* < 0.05). Key flavor compounds contributing distinct aromas included nonanal, octanal, benzaldehyde, hexanal, heptanal, 1-octen-3-ol, hexanol, pentanol, 2-heptanone, and 2-butanone. Among these, nonanal, octanal, benzaldehyde, hexanal, pentanol, and 1-octen-3-ol were present at significantly (*p* < 0.05) or highly significantly (*p* < 0.01) higher concentrations in the SH group compared to the others. Heptanal concentrations were highly significantly higher (*p* < 0.01) in both the SH and HH groups than in the NH group. Similarly, 2-butanone concentrations were highly significantly higher (*p* < 0.01) in the SH and HH groups compared to the NH and AH groups.

### 3.7. Classification of VOCs

Volatile flavor compounds play a crucial role in food, not only imparting unique aromas and tastes but also significantly enhancing the overall consumer eating experience. A total of 50 monomeric, dimeric, and trimeric compounds were identifiable across the four lamb groups. The monomers primarily consisted of the 8 aldehydes, 5 alcohols, 8 ketones, 2 esters, 1 acid, 1 furan, and 2 ethers mentioned earlier. While the types of volatile flavor compounds showed minimal variation across the four lamb groups, their concentrations exhibited significant differences. Specifically, the peak intensities of volatile compounds in the SH lamb were consistently higher than those observed in the AH, NH, and HH lambs. Higher concentrations of these volatile compounds correlate with more robust and full-bodied flavors. This enhanced flavor profile is likely to stimulate stronger consumer purchasing intent and aligns more readily with popular taste preferences.

Aldehydes play a pivotal role in the volatile flavor profile of lamb meat [48]. In this study, aldehydes constituted a high proportion of the identified volatile compounds. Aldehydes have been identified as key flavor compounds in the muscle tissues of ruminants and are decisive in forming the characteristic flavor of meat products. This is attributed to their extremely low flavor thresholds, meaning even minute quantities can impart intense aromas. Wang et al. conducted an analysis on indigenous Chinese sheep and found that aldehydes—specifically nonanal, octanal, heptanal, 3-methylbutanal, and hexanal—accounted for a significant proportion of the volatile compounds [49]. Our study found nonanal, octanal, and hexanal to be the most abundant aldehydes in the SH lamb. Hexanal, generated during the oxidation of lamb fat, imparts apple-like and fresh green leafy notes [50]. Octanal contributes grassy, fruity, and fatty notes. Nonanal imparts citrus and fatty notes [51]. Furthermore, the Strecker degradation reaction, a significant amino acid conversion pathway in organic chemistry, generates benzaldehyde from phenylalanine. Benzaldehyde, characterized by almond and caramel-like aromas, imparts a rich, appealing flavor to lamb, enhancing its flavor intensity and complexity [52].

The influence of alcohols on lamb flavor formation is less pronounced than that of aldehydes. However, they contribute significantly to the overall flavor profile in conjunction with aldehydes [53]. In muscle tissue, the degradation of linoleic acid is primarily catalyzed by the lipoxygenase and hydroperoxidase systems [54]. This biochemical process oxidatively cleaves unsaturated fatty acids, generating various aldehydes and alcohols. Major alcohols detected in lamb include hexanol, pentanol, ethanol, and 1-octen-3-ol. Among these, 1-octen-3-ol, derived from the oxidation of arachidonic acid and associated with mushroom, green, and vegetable aromas, significantly impacts lamb flavor formation due to its low threshold [51]. Pentanol possesses a fresh, oily nuance and can participate in Maillard reactions or esterification with fatty acids and amino acids, generating additional volatile flavor compounds that contribute to a richer and more complex lamb flavor profile [55].

Ketones typically originate from the oxidation or degradation of unsaturated fatty acids or may arise from Maillard reactions [54]. Most ketone compounds exhibit relatively high flavor perception thresholds [56]. As important products of lipid oxidative degradation, ketones are typically characterized by distinct fruity and creamy notes [53,57]. Within the volatile flavor composition of meat products, these compounds primarily contribute to the construction and balance of the overall flavor profile through synergistic effects [58]. Ketones can interact synergistically or antagonistically with other flavor components, significantly influencing the overall flavor profile of meat [59]. For instance, 2-heptanone imparts a cheesy note, while 2-butanone contributes a pleasant, fragrant aroma. 2-heptanone has been reported to play an important role in the volatile flavor of meat and meat products and is considered a marker compound for the characteristic flavor of lamb [13,60]. Among the four lamb groups analyzed, the SH lamb and HH lamb exhibited the highest levels of 2-butanone, enhancing the aroma intensity in these groups.

The primary synthetic pathways for ester compounds involve esterification reactions between organic acids (derived from fat or protein degradation) and alcohols, and transesterification reactions (alcoholysis) between fatty acids and alcohols, which can generate important products like triglycerides and ethanol [61].

Furans, characterized by meaty, broth-like, and roasted notes, contribute significantly [61]. 2-Pentylfuran and other furans originate from the Maillard reaction involving proteins and Strecker degradation. Research identifies 2-pentylfuran as one of the most important volatile compounds in meat [13]. Among the four lamb groups, the SH lamb contained the highest concentration of 2-pentylfuran, enriching the overall aromatic profile of the lamb. Most ether compounds emit pleasant aromas, adding complexity and depth to the flavor profile of meat products, making it more diverse.

### 3.8. Screening of Differential VOCs

In this study, we used the PLS-DA model to calculate VIP values, not for discriminative classification but to evaluate the contribution of each VOC variable to the overall differences. Different flavor compounds in mutton from different meat sheep breeds and their crossbred offspring with Hu sheep were screened based on variable importance for the projection (VIP). As shown in Table 3, using VIP > 1 as the criterion, a total of 10 differential flavor compounds were identified as potential biomarkers for distinguishing flavor profiles among the breeds. These compounds include Ethanol, (E)-2-Octenal, Cyclohexanone, Diallyl disulfide, Methyl isobutyl ketone-M, 1-Hexanol, Heptanal, (E, Z)-2,6-nonadienol, Benzaldehyde and Methyl isobutyl ketone-D.

The identified compounds play a significant role in shaping the overall flavor profile of meat. Aldehydes such as Heptanal and (E)-2-Octenal contribute fatty, green, and citrus notes. Diallyl disulfide offers a distinct garlic-like aroma, while Benzaldehyde introduces a characteristic almond nuance [62]. Alcohols including Ethanol and 1-Hexanol, along with ketones such as Cyclohexanone and Methyl isobutyl ketone, reflect underlying differences in lipid oxidation and breed-specific metabolism. Notably, (E, Z)-2,6-nonadienol, known for its cucumber-like scent, possesses an exceptionally low odor threshold, indicating its strong aromatic impact even at minimal concentrations [63]. Together, these biomarkers elucidate the molecular foundation of flavor variation among breeds, offering valuable insights into breed identification and meat quality assessment.

## 4. Conclusions

In conclusion, the NH hybrid lambs demonstrated superior meat quality overall. All four groups exhibited a rich array of amino acids and a balanced composition of essential amino acids, indicating that the lamb from each group serves as a high-quality protein source. Specifically, the NH group showed higher MUFA content and abundant total fatty acids, suggesting better meat quality and flavor, although it also contained two types of trans fatty acids at notably high levels. Meanwhile, the SH group displayed higher levels of volatile flavor compounds, contributing to a more robust and complex flavor profile. Based on these findings, this study recommends crossing SH sheep with HH sheep, improving slaughter performance and desirable flavor characteristics and indicating high economic potential for production.

## Figures and Tables

**Table 1:** Comparative analysis of the amino acid composition and content of lambs from the crosses between different breeds of mutton sheep and Hu sheep (ng/g).

Classification	Compound	HH	AH	SH	NH
Essential Amino Acids (EAAs)	Lys	11.59 ± 1.27 ^B^	11.69 ± 0.87 ^AB^	12.86 ± 2.34 ^AB^	13.93 ± 3.17 ^A^
	Met	1223.39 ± 119.80	1239.07 ± 126.68	1366.92 ± 217.20	1376.27 ± 415.46
	Thr	12.72 ± 2.06 ^A^	8.26 ± 2.18 ^B^	10.30 ± 4.51 ^A^	9.05 ± 1.48 ^A^
	Val	15.30 ± 2.50	15.52 ± 1.36	16.75 ± 4.24	16.66 ± 6.11
	Leu	4.79 ± 1.41	4.70 ± 1.28	5.22 ± 1.35	5.69 ± 1.65
	Ile	44.74 ± 6.73	44.73 ± 4.85	46.20 ± 8.63	47.71 ± 15.83
	Phe	621.60 ± 27.11	637.20 ± 26.09	645.01 ± 39.38	644.34 ± 46.39
Nonessential Amino Acids (NEAAs)	Trp	154.64 ± 56.00	136.78 ± 41.20	140.08 ± 69.67	109.53 ± 78.82
	Tyr	31.72 ± 3.45	31.81 ± 3.08	33.99 ± 4.35	33.03 ± 10.08
	Asp	375.24 ± 122.08 ^A^	272.52 ± 76.11 ^B^	319.43 ± 85.54 ^AB^	254.81 ± 67.93 ^B^
	His	6.13 ± 1.62 ^A^	4.86 ± 2.12 ^AB^	3.58 ± 0.79 ^B^	4.14 ± 0.78 ^B^
	Glu	155.14 ± 21.63 ^ab^	164.94 ± 39.59 ^a^	152.01 ± 23.50 ^ab^	135.41 ± 18.60 ^b^
	Gly	159.36 ± 53.36 ^a^	132.35 ± 16.40 ^ab^	119.71 ± 36.66 ^b^	134.70 ± 27.41 ^ab^
	Ala	45.74 ± 6.44	38.22 ± 5.17	44.10 ± 15.05	44.41 ± 8.59
	Arg	195.26 ± 19.26	199.38 ± 13.67	198.74 ± 29.23	215.23 ± 28.54
	Ser	44.68 ± 9.35	46.00 ± 4.24	48.25 ± 6.95	52.27 ± 15.19
	Creatine	248.05 ± 28.30	237.54 ± 22.80	235.58 ± 59.22	226.98 ± 25.32
	Pro	842.89 ± 186.94	864.40 ± 228.99	898.60 ± 171.73	904.47 ± 184.44
	Asn	27.11 ± 3.98	27.32 ± 2.31	27.76 ± 4.94	30.31 ± 8.34
	Gln	3.23 ± 0.65 ^ab^	4.52 ± 2.88 ^a^	2.49 ± 0.98 ^b^	3.93 ± 2.01 ^ab^
	GABAγ	35.23 ± 7.42 ^AB^	32.04 ± 8.49 ^B^	42.17 ± 5.33 ^A^	38.50 ± 8.73 ^AB^
	Orn	353.02 ± 112.88 ^A^	260.03 ± 75.00 ^AB^	304.35 ± 80.55 ^AB^	242.82 ± 64.17 ^B^
	Aminosulfonic acid	20.46 ± 4.14	18.48 ± 1.59	19.98 ± 5.93	20.42 ± 3.36
Total content of EAAs		2126.28 ± 170.89	2133.38 ± 171.68	2285.90 ± 271.91	2263.27 ± 493.25
Total content of NEAAs		2259.90 ± 231.59	2190.02 ± 190.66	2149.38 ± 188.32	2182.68 ± 227.76
Total content of TAAs		4386.18 ± 289.37	4323.39 ± 439.28	4435.29 ± 423.32	4445.96 ± 582.03
EAAs/TAAs		51.33 ± 1.57%	50.83 ± 1.72%	49.49 ± 1.90%	49.33 ± 2.11%
EAAs/NEAAs		94.62 ± 5.53%	96.62 ± 6.36%	98.84 ± 5.10%	98.60 ± 5.87%

Note: Data are expressed as means ± standard deviation (n = 3). Australian White–Hu (AH), White Suffolk–Hu (SH), Southdown–Hu (NH)—and a pure Hu sheep group (HH). Means with different superscript letters (a, b) are significantly different (*p* < 0.05); means with different superscript letters (A, B) are extremely significantly different (*p* < 0.01).

**Table 2:** Comparative analysis of the fatty acid composition and content of lambs from the crosses between different breeds of mutton sheep and Hu sheep (ng/g).

Classification	Compound	HH	AH	SH	NH
Saturated fatty acid (SFA)	Caprylic acid (C8:0)	310.84 ± 39.27	320.24 ± 95.61	293.30 ± 71.77	337.46 ± 55.15
	Decanoic acid (C10:0)	159.07 ± 99.67 ^B^	291.72 ± 256.47 ^AB^	213.00 ± 147.09 ^AB^	357.92 ± 105.32 ^A^
	Hendecanoic acid (C10:0)	28.29 ± 7.52 ^b^	34.37 ± 12.78 ^ab^	29.06 ± 11.42 ^b^	40.36 ± 3.87 ^a^
	Dodecanoic acid (C12:0)	1154.33 ± 298.20 ^AB^	1335.63 ± 598.93 ^AB^	1015.29 ± 278.05 ^B^	1450.10 ± 270.72 ^A^
	Tridecanoic acid (C13:0)	99.19 ± 37.66 ^ab^	98.23 ± 37.94 ^ab^	85.69 ± 26.05 ^b^	117.85 ± 24.26 ^a^
	Tetradecanoic acid (C14:0)	4692.60 ± 2043.43 ^B^	7659.88 ± 3620.96 ^AB^	5700.02 ± 3512.49 ^AB^	8605.97 ± 2616.60 ^A^
	Pentadecanoic acid (C15:0)	1427.23 ± 516.29	1790.42 ± 925.87	1569.02 ± 716.63	2000.98 ± 224.11
	Hexadecanoic acid (C16:0)	45,304.16 ± 14,146.01	54,161.89 ± 27,662.68	48,386.68 ± 18,798.95	60,515.64 ± 12,302.32
	cis-10-Heptadecenoic acid (C17:1)	2449.84 ± 995.45	2847.90 ± 1819.10	2873.79 ± 1415.98	3323.55 ± 639.52
	Octadecanoic acid (C18:0)	35,530.35 ± 11,317.99	38,974.76 ± 15,574.31	35,052.78 ± 11,084.17	42,937.20 ± 5021.36
	Arachidic acid (C20:0)	392.29 ± 72.67 ^AB^	401.19 ± 101.23 ^AB^	342.58 ± 57.36 ^B^	418.35 ± 81.27 ^A^
	Heneicosanoic acid (C21:0)	32.38 ± 6.91 ^ab^	32.44 ± 5.99 ^ab^	30.97 ± 3.94 ^b^	36.64 ± 7.10 ^a^
	Docosanoic acid (C22:0)	146.86 ± 2859.55 ^A^	124.43 ± 10.31 ^AB^	108.37 ± 11.23 ^B^	117.07 ± 11.29 ^B^
	Tetracosanoic acid (C24:0)	135.76 ± 28.40 ^B^	166.54 ± 41.74 ^A^	133.01 ± 13.59 ^B^	132.34 ± 10.69 ^B^
	SFA	93,178.20 ± 31,348.23	108,239.63 ± 52,365.65	95,833.56 ± 35,424.43	120,391.43 ± 20,488.42
Monounsaturated fatty acid (MUFA)	Myristoleic acid (C14:0)	506.55 ± 227.28 ^B^	901.74 ± 644.62 ^AB^	568.56 ± 342.33 ^B^	1058.80 ± 292.65 ^A^
	Myristelaidic acid (C14:1T)	339.72 ± 111.64 ^a^	282.22 ± 127.07 ^ab^	221.05 ± 38.17 ^b^	309.97 ± 103.05 ^ab^
	cis-10-Pentadecenoic acid (C15:1)	1319.99 ± 364.85 ^b^	1547.65 ± 612.46 ^ab^	1458.97 ± 618.81 ^ab^	1761.93 ± 145.28 ^a^
	trans-10-Pentadecenoic acid (C15:1T)	24.76 ± 15.85 ^B^	36.31 ± 20.81 ^AB^	15.45 ± 15.58 ^B^	42.61 ± 20.09 ^A^
	Palmitoleic acid (C16:1)	8258.75 ± 2768.82 ^B^	11,578.30 ± 7819.80 ^AB^	8375.74 ± 4575.75 ^B^	15,342.84 ± 3193.40 ^A^
	Palmitelaidic acid (C16:1T)	919.37 ± 365.77 ^B^	1289.57 ± 455.38 ^A^	1280.09 ± 484.01 ^A^	1339.03 ± 134.14 ^A^
	cis-10-Heptadecenoic acid (C17:1)	2123.28 ± 827.67 ^B^	2628.69 ± 1735.64 ^B^	2431.66 ± 1302.54 ^B^	3709.55 ± 311.14 ^A^
	trans-10-Heptadecenoic acid (C17:1T)	184.55 ± 82.88	209.67 ± 116.08	194.99 ± 86.99	247.57 ± 50.19
	Oleic acid (C18:1(n-9))	4057.71 ± 2228.07 ^b^	6493.13 ± 2678.27 ^a^	5524.31 ± 2497.64 ^ab^	5766.21 ± 594.90 ^ab^
	Petroselaidic acid (C18:1(n-12)T)	40,259.03 ± 14,589.45 ^b^	49,555.44 ± 27,034.61 ^ab^	44,606.10 ± 22,006.27 ^ab^	61,788.90 ± 9974.17 ^a^
	Elaidic acid (C18:1(n-9)T)	40,287.93 ± 14,265.10 ^b^	51,148.11 ± 28,797.41 ^ab^	45,229.43 ± 22,306.53 ^b^	63,570.22 ± 11,097.97 ^a^
	trans-Vaccenic acid (C18:1(n-7)T)	3772.76 ± 1916.31 ^b^	5763.92 ± 2514.67 ^a^	4949.46 ± 2169.14 ^ab^	5059.02 ± 620.23 ^ab^
	trans-7-Nonadecenoic acid (C19:1(n-12)T)	169.79 ± 63.31	170.90 ± 82.06	158.67 ± 83.68	179.07 ± 27.63
	trans-10-Nonadecenoic acid (C19:1(n-9)T)	9.41 ± 5.56 ^a^	8.84 ± 5.81 ^ab^	4.87 ± 2.64 ^b^	4.90 ± 3.32 ^b^
	cis-11-Eicosenoic acid (C20:1)	69.05 ± 26.42	83.58 ± 28.99	73.39 ± 18.14	77.30 ± 9.59
	trans-11-Eicosenoic acid (C20:1T)	575.41 ± 175.58 ^ab^	740.38 ± 386.62 ^ab^	550.78 ± 228.99 ^b^	797.51 ± 126.88 ^a^
	Brassidic acid (C22:1T)	64.32 ± 13.38 ^A^	54.21 ± 14.64 ^B^	47.97 ± 9.60 ^B^	53.67 ± 9.15 ^B^
	Nervonic acid (C24:1)	119.41 ± 33.08 ^A^	78.00 ± 22.64 ^B^	64.23 ± 17.06 ^B^	68.53 ± 7.90 ^B^
Polyunsaturated fatty acid (PUFA)	Linoleic acid (C18:2(n-6))	23,424.12 ± 8694.94	29,628.25 ± 11,734.93	30,340.17 ± 15,929.58	27,821.22 ± 3787.08
	Linoelaidic acid (C18:2(n-6)T)	353.87 ± 242.70	565.19 ± 306.95	400.12 ± 306.49	431.77 ± 268.91
	gamma-Linolenic acid (C18:3(n-6))	2184.22 ± 1236.70	2724.63 ± 1731.59	2594.17 ± 1479.69	3033.52 ± 943.23
	alpha-Linolenic acid (C18:3(n-3))	1629.13 ± 736.24	2447.73 ± 1184.31	2575.75 ± 1465.77	2338.78 ± 300.61
	cis-11,14-Eicosadienoic acid (C20:2)	388.81 ± 172.04 ^B^	466.74 ± 204.56 ^AB^	437.07 ± 169.94 ^AB^	502.35 ± 110.48 ^A^
	homo-gamma-Linolenic acid (C20:3(n-6))	2466.20 ± 1306.78	2623.86 ± 1346.38	2714.42 ± 1578.31	2948.34 ± 664.85
	cis-11,14,17-Eicosatrienoic acid (C20:3(n-3))	2392.70 ± 1330.47	2489.35 ± 1251.66	2620.31 ± 1514.03	2912.56 ± 566.86
	Arachidonic acid (C20:4)	69,538.43 ± 28,763.02	81,079.83 ± 39,663.67	78,261.97 ± 43,082.26	72,981.96 ± 10,706.60
	cis-13,16-Docosadienoic acid (C22:2)	99.27 ± 41.12 ^B^	120.63 ± 67.85 ^AB^	106.11 ± 60.7 ^AB^	148.38 ± 26.09 ^A^
	cis-5,8,11,14,17-Eicosapentaenoic acid (C20:5)	2403.81 ± 1069.46 ^b^	3025.84 ± 1447.54 ^ab^	3252.40 ± 655.89 ^ab^	3667.06 ± 1735.78 ^a^
	cis-7,10,13,16-Docosic acidtraenoic acid (C22:4)	5438.51 ± 1908.96	4695.10 ± 2164.36	4768.35 ± 2208.04	4963.75 ± 791.70
	cis-7,10,13,16,19-Docosapentaenoic acid (C22:5(n-3))	2918.64 ± 1046.13	3025.08 ± 1716.18	2586.29 ± 1621.37	2674.83 ± 426.37
	cis-4,7,10,13,16-Docosapentaenoic acid (C22:5(n-6))	3910.23 ± 1600.89	4130.92 ± 1480.73	4139.90 ± 2001.80	3604.90 ± 223.75
	cis-4,7,10,13,16,19-Docosahexaenoic acid (C22:6)	1271.65 ± 442.45	1709.82 ± 821.44	1523.66 ± 980.80	1309.63 ± 935.16
MUFA		102,882.60 ± 37,259.44 ^b^	132,390.93 ± 71,275.01 ^ab^	115,592.18 ± 55,959.04 ^b^	160,993.64 ± 24,799.08 ^a^
PUFA		118,598.78 ± 47,631.78	138,912.68 ± 63,408.65	136,484.23 ± 72,961.98	129,523.03 ± 17,067.12
Total content of fatty acid		314,659.57 ± 115,580.16 ^B^	379,543.24 ± 184,421.69 ^AB^	347,909.98 ± 163,870.85 ^AB^	410,908.09 ± 53,667.44 ^A^

Note: Data are expressed as means ± standard deviation (n = 3). Australian White–Hu (AH), White Suffolk–Hu (SH), Southdown–Hu (NH)—and a pure Hu sheep group (HH). Means with different superscript letters (a, b) are significantly different (*p* < 0.05); means with different superscript letters (A, B) are extremely significantly different (*p* < 0.01).

**Table 3:** Quantitative analysis by GC-IMS of volatile substances in lambs from the crosses between different breeds of mutton sheep and Hu sheep (mg/kg).

Compound	CAS	RI	RT/s	VIP	HH	AH	SH	NH
Nonanal-*M*	C124196	1105.5	799.763	<1	521.92 ± 163.59 ^B^	426.70 ± 124.94 ^B^	733.24 ± 113.74 ^A^	405.32 ± 119.35 ^B^
Nonanal-*D*	C124196	1105.9	800.721	<1	2664.76 ± 503.55 ^AB^	2396.90 ± 507.81 ^AB^	3110.73 ± 836.95 ^A^	2235.00 ± 461.53 ^B^
Octanal	C124130	1012.7	622.318	<1	1423.55 ± 283.14 ^B^	1559.11 ± 209.63 ^AB^	2018.54 ± 609.56 ^A^	1147.35 ± 322.49 ^B^
Benzaldehyde-*M*	C100527	961.4	520.589	1.05254	461.14 ± 79.06 ^ab^	492.82 ± 130.52 ^ab^	529.69 ± 34.27 ^a^	435.79 ± 71.42 ^b^
Benzaldehyde-*D*	C100527	960.7	519.333	<1	71.27 ± 10.18 ^C^	202.64 ± 32.36 ^B^	573.99 ± 61.56 ^A^	214.01 ± 88.38 ^B^
(E)-2-Heptenal	C18829555	959.5	516.821	<1	294.51 ± 90.06 ^AB^	267.17 ± 94.21 ^AB^	327.81 ± 79.90 ^A^	204.43 ± 71.90 ^B^
Heptanal-*M*	C111717	900	411.324	1.09563	2369.17 ± 662.23 ^A^	2100.28 ± 407.97 ^AB^	2281.19 ± 497.45 ^AB^	1440.26 ± 301.60 ^B^
Heptanal-*D*	C111717	900.4	411.952	<1	995.89 ± 138.48 ^B^	707.02 ± 273.82 ^B^	1322.82 ± 323.58 ^A^	316.14 ± 109.35 ^C^
Hexanal	C66251	791	277.569	<1	5494.14 ± 996.61 ^B^	4898.61 ± 502.42 ^B^	8527.65 ± 1125.68 ^A^	1845.56 ± 520.00 ^C^
Pentanal-M	C110623	689.6	188.247	<1	983.84 ± 331.25 ^AB^	712.86 ± 60.26 ^B^	1235.90 ± 485.69 ^A^	749.31 ± 161.25 ^B^
Pentanal-D	C110623	693	190.784	<1	242.69 ± 65.40 ^B^	187.33 ± 74.92 ^BC^	350.53 ± 74.55 ^A^	132.52 ± 27.05 ^C^
(E)-2-Octenal	C2548870	1066.3	719.414	1.52333	5241.23 ± 422.63 ^A^	5389.72 ± 497.43 ^A^	5625.52 ± 795.94 ^A^	4255.73 ± 552.56 ^B^
Octen-3-ol-*M*	C3391864	988.3	577.105	<1	2353.86 ± 526.65 ^AB^	1726.70 ± 320.23 ^B^	2581.33 ± 557.93 ^A^	1924.13 ± 374.77 ^AB^
Octen-3-ol-*D*	C3391864	986.3	572.709	<1	461.28 ± 178.09 ^B^	266.66 ± 88.77 ^C^	843.45 ± 57.95 ^A^	192.31 ± 66.05 ^C^
Octen-3-ol-*T*	C3391864	987.7	575.849	<1	73.85 ± 11.80 ^B^	76.02 ± 12.10 ^B^	78.49 ± 12.73 ^A^	71.35 ± 11.14 ^C^
(E,Z)-2,6-nonadienol	C28069729	1160.9	929.066	1.05271	1309.26 ± 244.02 ^A^	910.12 ± 108.25 ^B^	598.09 ± 30.24 ^C^	924.29 ± 259.07 ^B^
1-Hexanol-*M*	C111273	871.9	371.135	1.14100	1407.30 ± 382.68	1216.74 ± 271.99	1451.04 ± 364.60	1053.97 ± 473.46
1-Hexanol-*D*	C111273	871.4	370.507	<1	270.00 ± 144.59	194.17 ± 55.22	226.00 ± 64.16	201.85 ± 52.71
1-Entanol-*M*	C71410	765.2	251.822	<1	3345.46 ± 380.66 ^AB^	3140.12 ± 342.18 ^AB^	3820.31 ± 483.14 ^A^	2779.70 ± 728.45 ^B^
1-Entanol-*D*	C71410	762.6	249.31	<1	1412.10 ± 279.35 ^A^	979.99 ± 96.48 ^B^	1535.02 ± 71.72 ^A^	1303.81 ± 165.82 ^B^
Ethanol-*M*	C64175	474	109.686	1.94768	3272.39 ± 467.78 ^B^	1614.71 ± 211.81 ^C^	2905.57 ± 511.40 ^B^	3812.27 ± 404.50 ^A^
Ethanol-*D*	C64175	483.5	112.31	<1	11,402.77 ± 2569.81 ^A^	7898.11 ± 1380.52 ^B^	8650.22 ± 488.92 ^AB^	12,178.60 ± 4235.59 ^A^
Cyclohexanone-*M*	C108941	900	411.324	1.40992	387.53 ± 36.67 ^AB^	374.83 ± 74.47 ^AB^	434.91 ± 73.37 ^A^	344.80 ± 58.76 ^B^
Cyclohexanone-*D*	C108941	896.3	405.672	<1	232.71 ± 44.27 ^AB^	249.66 ± 66.99 ^AB^	268.05 ± 85.08 ^A^	177.02 ± 62.07 ^B^
2-Heptanone-*M*	C110430	890.5	396.881	<1	1128.25 ± 286.62	890.89 ± 143.85	1181.34 ± 356.50	944.16 ± 365.92
2-Heptanone-*D*	C110430	889.2	394.997	<1	356.34 ± 98.91	294.88 ± 63.68	355.25 ± 64.22	295.64 ± 64.89
2-Butanone-*M*	C78933	585.2	144.849	<1	2378.80 ± 205.61 ^A^	1948.90 ± 459.40 ^B^	2537.63 ± 304.86 ^A^	2222.57 ± 145.37 ^AB^
2-Butanone-*D*	C78933	585.2	144.849	<1	654.84 ± 136.69 ^ab^	538.68 ± 167.11 ^b^	724.09 ± 165.67 ^a^	568.04 ± 116.94 ^b^
2-Entanone-*M*	C107879	681.5	184.253	<1	1705.91 ± 294.57 ^AB^	1988.57 ± 479.15 ^A^	1342.76 ± 246.38 ^B^	1623.70 ± 124.72 ^AB^
2-Entanone-*D*	C107879	685.5	186.104	<1	5241.23 ± 422.63 ^A^	5389.72 ± 497.43 ^A^	5625.52 ± 795.94 ^A^	4255.73 ± 552.56 ^B^
1-Hydroxybutan-2-one-*M*	C513860	751.4	238.791	<1	3884.46 ± 294.49	3639.51 ± 539.58	3982.82 ± 384.63	3992.53 ± 442.65
1-Hydroxybutan-2-one-*D*	C513860	736.7	225.67	<1	9092.74 ± 1874.88 ^b^	9226.78 ± 3536.91 ^b^	11,692.70 ± 1266.95 ^a^	8840.16 ± 1181.37 ^b^
2-Hexanone	C626937	806	292.999	<1	4154.54 ± 776.79 ^ab^	3820.53 ± 560.39 ^b^	4444.47 ± 581.93 ^a^	3978.48 ± 778.94 ^b^
2-Octanone	C123966	1011.2	619.806	<1	386.78 ± 59.36 ^B^	346.55 ± 65.41 ^BC^	600.07 ± 111.27 ^A^	267.30 ± 43.13 ^C^
Methylisobutylketone-*M*	C108101	740.2	228.697	1.26386	517.40 ± 22.27 ^A^	549.49 ± 98.38 ^A^	420.30 ± 31.16 ^B^	525.50 ± 57.63 ^A^
Methylisobutylketone-*D*	C108101	738.9	227.549	1.05204	239.28 ± 88.31 ^a^	182.67 ± 39.04 ^b^	180.59 ± 45.98 ^b^	190.00 ± 21.69 ^b^
Ethyl 2-methylentanoate	C39255328	940.4	480.4	<1	199.15 ± 27.34	203.09 ± 50.66	177.48 ± 32.19	170.86 ± 42.37
Ethyl 2-hydroxyroanoate	C97643	820.8	308.967	<1	422.09 ± 84.11	388.70 ± 43.17	394.50 ± 47.75	394.12 ± 51.36
2-Methylbutanoicacid *-M*	C116530	870.4	369.251	<1	323.72 ± 34.08	328.93 ± 29.69	314.81 ± 26.05	323.38 ± 34.27
2-Methylbutanoicacid-*D*	C116530	871.9	371.135	<1	356.34 ± 98.91	294.88 ± 63.68	355.25 ± 64.22	295.64 ± 64.89
2-Pentylfuran	C3777693	996.5	595.689	<1	220.21 ± 106.08 ^ab^	147.28 ± 46.43 ^b^	289.97 ± 83.37 ^a^	174.89 ± 70.45 ^b^
1,8-Cineol	C470826	1028.4	649.321	<1	164.87 ± 48.72	131.78 ± 34.07	166.97 ± 56.49	145.90 ± 54.95
Diallyl disulfide	C2179579	1081.8	750.034	1.32708	422.06 ± 109.72	487.33 ± 45.27	442.23 ± 48.71	445.52 ± 50.95

Note: Means with different superscript letters (a, b) are significantly different (*p* < 0.05); means with different superscript letters (A, B, C) are extremely significantly different (*p* < 0.01).

## Data Availability

The original contributions presented in this study are included in the article. Further inquiries can be directed to the corresponding authors.
